# Peer Review of “Why We Are Losing the War Against COVID-19 on the Data Front and How to Reverse the Situation”

**DOI:** 10.2196/29418

**Published:** 2021-05-05

**Keywords:** COVID-19, learning health systems

*This is a peer-review report submitted for the paper “Why We Are Losing the War Against COVID-19 on the Data Front and How to Reverse the Situation”.*

## General Comments

This paper [1] is an important call to action for the health data community. We have a lot to learn about our failings in addressing this pandemic, and the authors highlight important and pragmatic holes. The authors would benefit from providing more concrete solutions. For example, rather than saying “we need standard data,” why not propose a data standard that can be universally adopted and modularly modified as needed? Rather, provide a list of key variables that everyone should include.

### Specific Comments

#### Major Comments

1. In the *What Can Be Done?* section, I suggest including a proposition for a data structure. We have time and time again called upon folks to collect data, just to be stuck with a bunch of disparate data that takes a heavy lift to integrate. If you do not have a specific structure, consider referring to an existing standard or calling upon the Centers for Disease Control and Prevention or Health Level Seven International to define a standard.

2. This paper lacks mention of existing companies, health systems, and government organizations that are already working on this. Highlighting existing initiatives will go a long way toward avoiding further data silos.

3. This paper is missing a key player in this work—electronic medical record vendors. They have the data, they know the data structures, and they have fewer checks and balances to data use than health systems do.
